# Analysis of Interrater Reliability and Interpretive Discrepancies in Polysomnography Scoring Across Clinical Subgroups

**DOI:** 10.3390/life16040669

**Published:** 2026-04-14

**Authors:** Ji Ho Choi, Tae Kyoung Ha, Ji Eun Moon, Seockhoon Chung

**Affiliations:** 1 Department of Otorhinolaryngology-Head and Neck Surgery, Soonchunhyang University Bucheon Hospital, Soonchunhyang University College of Medicine, 170, Jomaru-ro, Bucheon 14584, Republic of Korea; 2 HoneyNaps USA, Inc., #517, SPACES, 361 Newbury Street, Boston, MA 02115, USA; 3 Graduate School (Biostatistics Section), Chungwoon University, 113, Sukgol-ro, Michuhol-gu, Incheon 22100, Republic of Korea; 4 Department of Psychiatry, Asan Medical Center, University of Ulsan College of Medicine, 86, Olympic-ro 43-gil, Songpa-gu, Seoul 05505, Republic of Korea

**Keywords:** interrater reliability, polysomnography, sleep stages, respiratory events, scoring

## Abstract

Background: Polysomnography (PSG) is the gold standard for diagnosing sleep disorders. However, the subjectivity of manual scoring can lead to inter-scorer variability, undermining diagnostic accuracy and subsequent clinical decisions. This study aims to quantitatively assess scoring concordance among multiple scorers across various clinical subgroups to identify the factors that contribute to interpretive discrepancies. Methods: We conducted a retrospective analysis of overnight diagnostic PSG data from adult patients at a tertiary university hospital sleep center. Interrater reliability was evaluated by three independent expert scorers for 30 subjects selected through stratified random sampling. The polysomnographic data were independently and blindly scored according to the American Academy of Sleep Medicine criteria, focusing on sleep stages, arousals, respiratory events, and leg movements, all scored in 30 s epochs. Interrater agreement was measured using Fleiss’ κ, along with 95% confidence intervals, and included subgroup analyses by diagnostic category. Results: The analysis included a total of 28,291 epochs from 30 adults across normal, insomnia, obstructive sleep apnea (OSA) [mild–severe], and periodic limb movement (PLM) disorder subgroups. The overall interrater agreement for sleep staging among the three scorers was nearly perfect (Fleiss’ κ = 0.932), with the highest concordance observed in stages W, N2, and R, and excellent agreement in stages N1 and N3. Respiratory events showed particularly high reliability, with near-perfect agreement for apnea (κ = 0.955) and substantial agreement for hypopnea, arousals, and PLMs. Pairwise analyses indicated the highest concordance between scorer 1 and scorer 3, while the agreement between scorer 1 and scorer 2 was lower, particularly for detecting arousals and limb movements. Subgroup analyses showed the highest and most stable agreement in moderate OSA, whereas severe OSA exhibited reduced reliability for sleep staging and arousal scoring, indicating increased scoring complexity with greater sleep fragmentation. Conclusions: Although expert PSG scoring demonstrates high overall reliability, significant variability persists in complex cases like severe OSA. These findings underscore the necessity for structured quality assurance and automated tools to improve diagnostic consistency in clinical practice.

## 1. Introduction

Polysomnography (PSG) is the established gold standard for diagnosing and evaluating a wide range of sleep disorders, particularly obstructive sleep apnea (OSA) and periodic limb movement (PLM) disorder (PLMD) [1,2,3]. As a sophisticated multi-channel diagnostic tool, PSG allows for the simultaneous and continuous monitoring of various physiological parameters, including electrooculography (EOG), electroencephalography (EEG), electrocardiography (ECG), chin and leg electromyography (EMG), airflow, respiratory efforts, oxygen saturation, snoring, and sleep position. This comprehensive monitoring enables clinicians to accurately delineate sleep architecture and identify complex pathological events [4,5]. The careful scoring of sleep stages, arousals, and respiratory events (such as apneas and hypopneas) is crucial, as these metrics not only enhance diagnostic accuracy but also inform personalized treatment strategies and predict long-term clinical outcomes [6,7,8].

Despite being based on high-resolution physiological signals, the scoring of PSG is primarily a manual process that requires expert interpretation [9,10]. To ensure consistency and reduce inter-scorer variability, the American Academy of Sleep Medicine (AASM) has developed standardized scoring manuals that outline detailed criteria for assessing sleep architecture and respiratory events [11,12,13]. However, in real-world clinical settings, several confounding factors—such as the scorer’s clinical expertise, individual interpretive judgment, and external influences like shift timing and cognitive fatigue—can affect the scoring process [14,15,16,17]. These variables introduce a degree of subjectivity, which may compromise the reliability and reproducibility of diagnostic results. This subjectivity is evident in inter-scorer variability, a significant source of inconsistency in both clinical diagnosis and research data.

Discrepancies among scorers in sleep stage scoring often arise due to several confounding factors, including physiological artifacts, variations in signal quality, and differing interpretations of transitional or ambiguous sleep epochs [12,14,18,19,20]. Recent meta-analyses have consistently shown that interrater reliability in PSG scoring varies significantly across different sleep stages [21]. While the overall Cohen’s κ for sleep stage scoring is approximately 0.76—considered ‘substantial agreement’—the concordance level for Stage N1 is notably lower than for other stages [21]. This discrepancy is largely due to Stage N1’s ambiguous physiological characteristics and the absence of clear signal boundaries. These findings highlight that, despite standardized scoring guidelines, interpretive deviations remain for specific sleep stages and events, underscoring the ongoing need for rigorous scorer training and refinement of scoring criteria.

Quantitatively assessing the degree of concordance among multiple scorers interpreting the same polysomnographic data in a clinical setting is crucial. A thorough analysis of interrater reliability not only validates the objectivity of assessments but also identifies factors that contribute to reduced agreement in specific stages or events. It serves as a foundation for refining scoring criteria and establishes a benchmark for developing automated PSG analysis algorithms. Therefore, this study aims to quantitatively validate the reliability of PSG scoring from the perspective of multiple evaluators. To accomplish this, we will calculate the interrater reliability among three experienced scorers who independently interpreted identical polysomnographic records, focusing on key parameters such as sleep stages (Stage W, N1, N2, N3, and rapid eye movement [REM]), arousals, respiratory events (apneas and hypopneas), and limb movements. Additionally, by including a diverse cohort of 30 participants—comprising a healthy control group and various clinical subgroups with sleep disorders such as insomnia, mild-to-severe OSA, and PLMD—this study aims to determine whether significant variations in scoring concordance exist across different clinical subgroups.

## 2. Materials and Methods

### 2.1. Subjects

This study was carefully designed to evaluate interrater reliability by having three independent scorers analyze the same retrospectively collected PSG datasets. The study involved adult patients who underwent overnight diagnostic PSG at a tertiary university hospital sleep center between December 2016 and December 2019. Eligibility was limited to adults aged 20 years and older. Exclusion criteria included patients diagnosed with or concurrently suffering from sleep disorders other than OSA, insomnia, and PLMD, as well as cases with inaccessible, illegible, or insufficient medical records for thorough analysis. Using a stratified random sampling process, 30 participants were selected and categorized into the following subgroups: Normal (*n* = 8), Insomnia (*n* = 2), Mild OSA (*n* = 5), Moderate OSA (*n* = 5), Severe OSA (*n* = 5), and PLMD (*n* = 5). The “Normal” group consisted of individuals with no clinically significant sleep disorders. The “Insomnia” group included cases with subjective complaints and clinical signs of difficulty initiating or maintaining sleep, or early morning awakening. Subgroups were classified based on the apnea-hypopnea index (AHI): mild OSA (5 ≤ AHI < 15), moderate OSA (15 ≤ AHI < 30), and severe OSA (AHI ≥ 30). The PLMD group was defined by a PLM index ≥ 15. All diagnostic classifications adhered strictly to the criteria outlined in the International Classification of Sleep Disorders, Third Edition. The study protocol received formal review and approval from the Institutional Review Board (IRB) of Soonchunhyang University Bucheon Hospital (IRB No. 2025-12-022). In alignment with ethical standards and institutional guidelines for retrospective data analysis, the requirement for obtaining written informed consent was waived.

### 2.2. Polysomnography

All participants underwent overnight PSG following a standardized protocol with a Level 1 comprehensive diagnostic system (Embla N7000; Natus Medical Inc., San Carlos, CA, USA). The recording montage utilized a multi-channel configuration to ensure high-quality data acquisition, including EEG, EOG, chin and leg EMG, ECG, pulse oximetry, nasal pressure transducers, and thoracic/abdominal respiratory inductive plethysmography belts. EEG signals were collected from at least six channels according to the international 10–20 system to enable accurate sleep staging. EOG and EMG electrodes were placed to monitor bilateral eye movements and submental muscle activity, respectively. Airflow was monitored using both a nasal pressure transducer and a thermal sensor to accurately detect respiratory events such as apneas and hypopneas, providing qualitative and quantitative respiratory data. Additionally, bilateral anterior tibialis EMG recordings were taken to identify and quantify PLMs. To ensure data integrity and consistency across all participants, a uniform sampling rate and standardized filtering criteria—specific high-pass and low-pass filters for each signal type—were rigorously applied to all polysomnographic recordings.

### 2.3. Scoring

The scoring process involved three highly skilled experts: two registered polysomnographic technologists (RPSGT) and one physician certified as an International Sleep Specialist (intl. ABSM, USA). To maintain interpretive consistency, all scorers were required to have at least 10 years of professional experience in PSG scoring. Before the study began, they participated in a thorough review and training session on the latest AASM manual for scoring sleep and related events (Version 3.0) to align their interpretive criteria and minimize discrepancies [11]. To eliminate information bias and ensure objectivity, the scoring was conducted in a blinded manner. The evaluators received no additional information, including clinical diagnoses, patient histories, or the scoring results from other scorers. This independent protocol ensured that each scoring outcome was based solely on the physiological data, thereby enhancing the internal validity of the interrater reliability analysis. Sleep architecture and associated events were systematically coded for each 30 s epoch. All scorers followed a standardized protocol in line with the latest AASM scoring manual to ensure methodological rigor. Sleep stages were classified as nominal categorical data and assigned to one of five distinct stages: wakefulness (W), REM sleep (R), and non-REM (N) sleep stages N1, N2, and N3. Physiological events such as arousals, apneas, hypopneas, and limb movements were meticulously quantified within each epoch. To maintain consistency across all evaluators, a specific temporal rule was applied for events spanning multiple epochs: events were recorded in the epoch containing their onset or the physiologically dominant portion. This systematic approach eliminated ambiguity in event allocation and facilitated uniform analysis of event frequencies. All evaluators underwent a dedicated orientation and training session to ensure a comprehensive understanding and strict adherence to these coding procedures, reinforcing the overall reliability of the data collection process.

### 2.4. Statistics

Interrater agreement among the three independent scorers was quantitatively assessed using Fleiss’ κ, a well-established statistic for evaluating agreement among multiple scorers beyond chance. Fleiss’ κ was chosen a priori as the primary measure of reliability and was calculated for overall sleep staging as well as for specific scoring parameters, including the various sleep stages (W, N1, N2, N3, and R), arousals, apneas, hypopneas, and periodic leg movements. For each analysis, 95% confidence intervals (CIs) were estimated to quantify the precision of the κ estimates. The magnitude of agreement was interpreted using the criteria proposed by Landis and Koch, which categorize κ values as poor (<0.00), slight (0.00–0.20), fair (0.21–0.40), moderate (0.41–0.60), substantial (0.61–0.80), or almost perfect (0.81–1.00) [22,23]. To assess the robustness of interrater reliability across varying levels of diagnostic complexity, prespecified subgroup analyses were conducted by comparing κ estimates across different clinical disease clusters. Statistical analyses were performed using Rex (Version 3.0.3; RexSoft Inc., Seoul, Republic of Korea; https://rexsoft.org/).

## 3. Results

A total of 28,291 epochs were derived from 30 subjects (17 males and 13 females) with a mean age of 44.9 ± 12.1 years. The dataset included a range of clinical conditions, distributed as follows: normal (*n* = 8, 7722 epochs), insomnia (*n* = 2, 1999 epochs), mild OSA (*n* = 5, 4920 epochs), moderate OSA (*n* = 5, 4359 epochs), severe OSA (*n* = 5, 4443 epochs), and PLMD (*n* = 5, 4848 epochs).

The interrater reliability for sleep staging and respiratory/movement events among the three scorers is summarized in Table 1. The analysis of overall sleep staging yielded a Fleiss’ κ coefficient of 0.932 (95% CI 0.928–0.936), indicating “almost perfect agreement” among the scorers. In a detailed sub-analysis of each sleep stage, Stage W exhibited the highest level of concordance with a κ of 0.972 (95% CI 0.966–0.977), followed by Stage N2 (κ = 0.934, 95% CI 0.928–0.939) and Stage R (κ = 0.933, 95% CI 0.927–0.938). Conversely, Stage N1 and Stage N3 displayed relatively lower reliability compared to other stages, both recorded at κ = 0.867 (95% CI 0.861–0.872); however, these values still indicate an “excellent” level of statistical agreement. For arousal events, the κ coefficient was 0.869 (95% CI 0.863–0.875). In the analysis of respiratory events, apnea showed the highest reliability among all examined variables, with a κ of 0.955 (95% CI 0.949–0.961). Hypopnea also demonstrated substantial reliability, with κ = 0.929 (95% CI 0.923–0.935). Lastly, PLM was associated with a κ value of 0.867 (95% CI 0.861–0.873).

Inter-rater reliability for sleep variables is summarized in Table 2. Overall, the pair of Raters 1 and 3 consistently demonstrated the highest agreement across most variables, while the pair of Raters 1 and 2 tended to show the lowest. For sleep stage scoring, the highest agreement was observed between Raters 1 and 3 (κ = 0.974; 95% CI: 0.967–0.981), whereas Raters 1 and 2 showed the lowest (κ = 0.898; 95% CI: 0.891–0.905). Notably, all rater pairs achieved “excellent” agreement (κ ≥ 0.89) for this category. In the case of arousal events, the Rater 1 vs. 2 pair recorded a relatively lower agreement (κ = 0.804) compared to other variables, while the Rater 1 vs. 3 (κ = 0.906) and Rater 2 vs. 3 (κ = 0.897) pairs exhibited high reliability near 0.90. Regarding respiratory events, apnea scoring showed high consistency across all pairs (κ ≥ 0.93), peaking between Raters 1 and 3 (κ = 0.972). Similarly, hypopnea scoring reached its highest agreement between Raters 1 and 3 (kappa = 0.963); although the Rater 1 vs. 2 pair showed the lowest agreement (κ = 0.896), it still indicated high reliability. PLM scoring followed a different trend, with the highest agreement found between Raters 2 and 3 (κ = 0.922; 95% CI: 0.912–0.932). The lowest overall κ value across all variables was recorded between Raters 1 and 2 for PLM scoring (κ = 0.791).

The interrater reliability for key polysomnographic parameters across different sleep disorder subgroups is presented in Table 3. The analysis demonstrated strong reliability for most variables, with κ coefficients consistently exceeding 0.80. Notably, the moderate OSA group exhibited the highest and most stable concordance across all examined parameters. In terms of sleep staging, the highest levels of agreement were observed in the moderate OSA (κ = 0.954) and PLMD (κ = 0.951) groups. In contrast, the severe OSA group yielded the lowest κ value of 0.859, suggesting that increased sleep fragmentation in severe OSA patients may enhance the subjectivity of sleep stage scoring. A similar trend was observed for arousal detection, with the moderate OSA group achieving a peak κ of 0.934, whereas the severe OSA group showed relatively lower agreement (κ = 0.823). Regarding respiratory events, the moderate OSA group demonstrated near-perfect agreement for apnea (κ = 0.990), with high reliability also maintained in the severe (κ = 0.941) and mild (κ = 0.923) OSA groups. Hypopnea scoring followed a similar pattern, peaking in the moderate OSA group (κ = 0.980) and followed by the mild (κ = 0.900) and severe (κ = 0.885) OSA groups. Finally, for the PLMD subgroup, the reliability for PLM scoring was identified as 0.870 (95% CI 0.856–0.884), indicating a substantial level of consensus.

Table 4 details the pairwise interrater reliability for key polysomnographic parameters across clinical subgroups. Consistent with the overall findings, the pairing of scorer 1 and scorer 3 exhibited the highest concordance for most variables, while the scorer 1–2 pair generally showed the lowest agreement. Apnea scoring was the most reliable parameter, maintaining a high reliability (κ ≥ 0.90) across all subgroups and scorer combinations. The moderate OSA group was the most consistently scored cohort, with κ values exceeding 0.90 for all variables and scorer pairs. Notably, the agreement for sleep staging in this group peaked between scorer 1 and scorer 3 (κ = 0.974, 95% CI 0.956–0.991). In contrast, the severe OSA group posed the greatest challenge for interrater consistency. Specifically, the scorer 1–2 pair recorded the lowest agreement for sleep staging (κ = 0.787) and arousal detection (κ = 0.735) in this subgroup. These lower scores suggest that the fragmented sleep architecture typical of severe OSA complicates the scoring process significantly. For PLMD patients, the scoring of PLMs was most consistent between scorer 2 and scorer 3 (κ = 0.909), while the scorer 1–2 pair demonstrated a lower concordance of 0.789.

## 4. Discussion

The present study provided a rigorous quantitative assessment of interrater reliability in scoring sleep stages and respiratory/movement events by analyzing PSG data from a diverse cohort of 30 participants, which included six distinct clinical subgroups: normal, insomnia, and mild, moderate, and severe OSA, as well as PLMD. An analysis of the extensive dataset, comprising a total of 28,291 epochs, revealed an overall Fleiss’ κ coefficient of 0.932 for sleep staging, indicating “almost perfect agreement” among the three expert scorers. This exceptionally high level of concordance emphasizes that significant objectivity and diagnostic consistency can be achieved in clinical practice when experienced evaluators rigorously adhere to the standardized scoring criteria set forth by the latest sleep medicine guidelines.

Analysis of individual sleep stages revealed that stage W exhibited the highest level of concordance (κ = 0.972), primarily due to the distinct and easily identifiable EEG characteristics of wakefulness compared to other stages. Stage N2 (κ = 0.934) and stage R (κ = 0.933) also demonstrated exceptional reliability. This high level of agreement indicates that pathognomonic features—such as K-complexes and sleep spindles in stage N2, and REMs accompanied by low-amplitude, mixed-frequency EEG patterns in stage R—served as clear landmarks for the scorers. In contrast, stage N1 and stage N3 showed relatively lower, though still substantial, agreement (κ = 0.867). The reduced concordance in stage N1 likely arises from its transitional nature between wakefulness and sleep, where subtle shifts in EEG frequencies allow for greater subjective interpretation. For stage N3, discrepancies seem to stem from slight temporal variations in measuring the amplitude and percentage of slow-wave activity, which can lead to differing epoch classifications among scorers. These findings are consistent with existing literature. A recent meta-analysis on interrater reliability in sleep stage scoring reported that Cohen’s κ values were highest for stages W and R, while stage N1 consistently exhibited the lowest agreement [21]. Similarly, the 2013 AASM inter-scorer reliability program identified a hierarchy of agreement, with stages R, N2, and W showing the highest concordance, whereas stages N1 and N3 proved to be the most challenging for scorers [12]. Notably, all sleep stages in the present study achieved κ values exceeding 0.80—a threshold indicating “almost perfect” agreement—demonstrating the high level of expertise among the evaluators.

Among the respiratory events, apnea showed the highest level of concordance (κ = 0.955) across all analyzed variables. This strong agreement is likely due to the clear visual evidence of airflow cessation, which minimizes subjective interpretation among evaluators. Hypopnea also demonstrated a robust κ of 0.929, indicating that scorers had a highly synchronized understanding and application of the standardized hypopnea criteria [11]. In contrast, the concordance for arousals (κ = 0.869) and PLMs (κ = 0.867) was relatively lower. Identifying arousals requires detecting transient EEG frequency shifts lasting at least three seconds, a task that demands intense focus and is prone to inter-scorer variability. Similarly, scoring PLMs involves precise measurements of EMG burst duration and inter-movement intervals, where attentional fluctuations and subjective judgment can play a larger role. Notably, the pairwise agreement for PLMs between scorer 1 and scorer 2 was the lowest at 0.791, suggesting that subtle individual variations in interpreting limb movement guidelines may persist even among experienced professionals.

One of the most pivotal findings of this study is the significant variance in scoring reliability observed across clinical subgroups. The moderate OSA group exhibited the most stable and consistently high concordance across nearly all parameters, including sleep staging (κ = 0.954), apnea (κ = 0.990), and hypopnea (κ = 0.980). This suggests that moderate respiratory impairment may present the most “textbook” or prototypical physiological patterns, providing scorers with clear diagnostic signals that minimize interpretive errors. In contrast, the severe OSA group demonstrated the lowest levels of agreement, particularly in sleep staging (κ = 0.859) and arousal detection (κ = 0.823). This diminished reliability is likely a consequence of extreme sleep fragmentation and recurrent arousals characteristic of severe sleep-disordered breathing [24]. Such pathological conditions disrupt the continuity of EEG signals and induce frequent, abrupt transitions between sleep stages, maximizing interpretive ambiguity. Notably, the interrater agreement for sleep staging between a specific pair of scorers (scorer 1 vs. 2) dropped to 0.787 in the severe OSA subgroup, underscoring a critical clinical implication: the interpretation of PSG data for patients with high-severity disorders is inherently more complex and necessitates more rigorous cross-validation or consensus-based scoring to ensure diagnostic precision.

In the PLMD subgroup, interrater reliability was generally high across the evaluated parameters, with κ values of 0.951 for sleep stage, 0.887 for arousal, and 0.870 for periodic leg movements. Pairwise analyses similarly indicated consistent agreement among scorers, supporting the overall reproducibility of scoring within this group. These findings suggest that periodic leg movements, which often exhibit relatively stereotyped patterns, can be identified with reasonable consistency using standard criteria. However, the slightly lower agreement for periodic leg movements compared to sleep staging indicates that some variability remains in scoring these events. This residual variability may reflect differences in interpreting the onset and termination of movements or subtle variations in signal presentation [3,24]. Therefore, while the PLMD subgroup demonstrates stable scoring performance overall, caution is warranted when interpreting movement-related indices, as inter-scorer differences may still influence results.

Based on the pairwise interrater analysis (Table 2 and Table 4), the level of agreement between scorer 1 and scorer 3 was generally the highest, while the concordance between scorer 1 and scorer 2 was relatively lower. These deviations among individual scorer pairs underscore the potential risks in clinical environments where diagnostic outcomes may rely on a single evaluator’s interpretation. Notably, the agreement for arousal scoring between scorers 1 and 2 yielded a Fleiss’ κ of 0.804, demonstrating that individual variations in sensitivity—specifically in detecting subtle EEG shifts—can significantly influence overall scoring consistency. These findings suggest that personal interpretive thresholds for identifying micro-arousals may differ even among experts, highlighting the need for continuous cross-training and institutional quality control to mitigate the impact of individual diagnostic subjectivity.

To enhance and sustain high inter-scorer reliability in PSG, a multi-faceted approach that includes educational, systemic, and technological strategies is essential. First, implementing standardized quality management programs and conducting regular inter-scorer reliability monitoring is crucial. Periodic systematic testing, where all evaluators score identical sample datasets to assess concordance through appropriate statistical analyses, should be established. When significant discrepancies occur, consensus meetings should be held to review differing epochs and re-establish interpretive standards. Additionally, intensive training on the latest AASM guidelines is vital to align the sensitivity in identifying complex signals, such as micro-arousals, thereby reducing individual subjective bias. Second, adopting multiple scoring systems and cross-validation protocols can help minimize the risks associated with single-scorer dependency. This can be accomplished through random cross-checks, where a specified percentage of total data is re-evaluated by a second scorer to identify personal bias. For clinical subgroups with lower concordance—such as those with severe OSA or PLMD—a joint review or dual-approval system should be implemented to ensure diagnostic accuracy. Third, integrating artificial intelligence (AI)-based automated PSG analysis presents a robust solution to human variability. By leveraging AI algorithms, inconsistencies arising from a scorer’s physical condition or subjectivity can be reduced. Employing AI for initial screening and using it as a conflict-detection tool—where the system flags epochs with significant human-AI discordance for manual re-evaluation—can enhance diagnostic accuracy in clinical laboratories. While this study primarily focuses on interrater reliability among human experts, the role of AI technology warrants more in-depth discussion. In the current landscape, high-quality human-scored data remain the essential “ground truth” required to train and validate AI algorithms. Our findings, particularly the identification of specific interpretive discrepancies in complex cases like severe OSA, provide critical benchmark data that can guide the future development and refinement of AI-based scoring tools. Finally, standardizing the scoring environment and managing cognitive fatigue are critical for maintaining interpretive quality. Technical variables, such as monitor resolution, signal filters, and software interfaces, must be consistent across all workstations to eliminate equipment-related variance. Additionally, institutional policies should limit the daily volume of manual scoring and mandate adequate rest periods, as cognitive exhaustion significantly impacts the ability to detect subtle EEG fluctuations. In summary, while recognizing individual differences in scorer sensitivity, a comprehensive strategy that integrates systemic cross-validation, data-driven education, and AI-assisted technologies represents the most effective safeguard against diagnostic errors in clinical settings.

This study presents meaningful findings, but it has several limitations. First, the total sample size was limited to 30 participants with an imbalanced distribution across clinical subgroups, which may restrict the generalizability of our results. Nevertheless, as an exploratory analysis, our detailed assessment of a large number of epochs (28,291) supports the internal consistency and reliability of the observed scoring trends. Consequently, the findings, particularly those related to smaller clinical subgroups, should be interpreted with caution. Second, the exceptionally high interrater agreement (κ > 0.93) may reflect an idealized upper bound rather than routine clinical variability, as all scorers were highly experienced experts with over 15 years of expertise. Furthermore, this high degree of consensus may be attributed to the scorers’ shared institutional background and their regular participation in standardized quality management programs; while ensuring high internal consistency, such alignment may limit the external validity of the findings in diverse clinical settings. Third, this investigation involved evaluations by only three scorers. While this is an improvement over traditional two-scorer studies, it raises concerns about the potential overestimation of interrater reliability. If the scorers have similar educational backgrounds, clinical training, or institutional affiliations, their concordance may be artificially high compared to what is typically observed across diverse clinical settings. Finally, our analysis mainly focused on quantifying numerical agreement among scorers, without exploring the practical clinical implications of scoring discrepancies. We did not examine how disagreements might affect clinical diagnoses or therapeutic strategies. Therefore, future research should involve larger, more diverse cohorts with a balanced distribution of sleep disorders. Large-scale prospective studies are needed to thoroughly assess how inter-scorer variability impacts clinical decision-making and patient management outcomes.

## 5. Conclusions

This study quantitatively demonstrated the high reliability of PSG scoring by analyzing a comprehensive dataset of 28,291 epochs from 30 patients, interpreted by three expert scorers. Our findings yielded an overall Fleiss’ κ of 0.932 for sleep staging, indicating “almost perfect agreement” among professionals. However, we observed significant variations in reliability based on the clarity of scoring criteria for specific parameters. Notably, the severe OSA subgroup showed the lowest concordance due to pronounced sleep fragmentation and frequent stage transitions. This underscores the need for more rigorous validation protocols when interpreting PSG data from high-risk clinical populations. The discrepancies in individual sensitivity and pairwise agreement highlight the potential for diagnostic errors inherent in single-scorer interpretations in clinical settings. To address these issues, it is essential to implement standardized quality management programs for inter-scorer reliability and to conduct regular consensus meetings to reconcile subjective interpretive differences. Additionally, establishing systemic safeguards, such as multiple scoring systems and random cross-validation, is crucial for reducing the influence of individual bias on final diagnostic and therapeutic decisions. Prioritizing the integration of AI-based automated scoring technologies as a supplementary tool can help mitigate variability caused by human subjectivity and fatigue. It is also important to standardize the scoring environment and regulate workload to prevent cognitive exhaustion from impairing the detection of subtle neurophysiological changes. Despite limitations related to sample size and subgroup distribution, this study offers meaningful, actionable strategies for enhancing diagnostic reliability through a data-driven approach. Ultimately, maximizing the objectivity of sleep diagnostics requires a combination of systemic refinement and technological innovation. Future research should focus on how scoring discordance directly affects long-term clinical outcomes.

## Figures and Tables

**Table 1:** Interrater reliability (Fleiss’ κ) for scoring sleep stages, arousals, and respiratory and movement events among three raters.

Variables	κ (95% Confidence Intervals)
Sleep stage	0.932 (0.928–0.936)
Stage W	0.972 (0.966–0.977)
Stage N1	0.867 (0.861–0.872)
Stage N2	0.934 (0.928–0.939)
Stage N3	0.867 (0.861–0.872)
Stage R	0.933 (0.927–0.938)
Arousal	0.869 (0.863–0.875)
Apnea	0.955 (0.949–0.961)
Hypopnea	0.929 (0.923–0.935)
Periodic leg movement	0.867 (0.861–0.873)

**Table 2:** Pairwise interrater reliability (Fleiss’ κ) of polysomnography scoring parameters.

Variables	κ (95% Confidence Intervals)		
	1 vs. 2	1 vs. 3	2 vs. 3
Sleep stage	0.898 (0.891–0.905)	0.974 (0.967–0.981)	0.924 (0.917–0.931)
Arousal	0.804 (0.792–0.815)	0.906 (0.894–0.918)	0.897 (0.885–0.909)
Apnea	0.932 (0.920–0.944)	0.972 (0.960–0.984)	0.960 (0.948–0.972)
Hypopnea	0.896 (0.884–0.907)	0.963 (0.951–0.975)	0.930 (0.918–0.942)
Periodic leg movement	0.791 (0.781–0.801)	0.880 (0.870–0.890)	0.922 (0.912–0.932)

**Table 3:** Interrater reliability (Fleiss’ κ) of sleep parameters across different subgroups.

Subgroup (Number of Epochs)	Variables	κ (95% Confidence Intervals)
Normal (*n* = 7722)	Sleep stage	0.944 (0.936–0.952)
	Arousal	0.858 (0.846–0.870)
Insomnia (*n* = 1999)	Sleep stage	0.969 (0.951–0.986)
	Arousal	0.868 (0.842–0.893)
Mild OSA (*n* = 4920)	Sleep stage	0.926 (0.916–0.936)
	Arousal	0.847 (0.831–0.863)
	Apnea	0.923 (0.907–0.939)
	Hypopnea	0.900 (0.884–0.915)
Moderate OSA (*n* = 4359)	Sleep stage	0.954 (0.944–0.963)
	Arousal	0.934 (0.918–0.950)
	Apnea	0.990 (0.972–1.000)
	Hypopnea	0.980 (0.964–0.995)
Severe OSA (*n* = 4443)	Sleep stage	0.859 (0.849–0.869)
	Arousal	0.823 (0.807–0.839)
	Apnea	0.941 (0.925–0.957)
	Hypopnea	0.885 (0.869–0.901)
Periodic leg movement disorder (*n* = 4848)	Sleep stage	0.951 (0.941–0.961)
	Arousal	0.887 (0.871–0.903)
	Periodic leg movement	0.870 (0.856–0.884)

OSA, obstructive sleep apnea.

**Table 4:** Pairwise interrater reliability (Fleiss’ κ) of sleep parameters across different subgroups.

Subgroup (Number of Epochs)	Variables	κ (95% Confidence Intervals)		
		1 vs. 2	1 vs. 3	2 vs. 3
Normal (*n* = 7722)	Sleep stage	0.916 (0.902–0.930)	0.975 (0.961–0.989)	0.942 (0.928–0.956)
	Arousal	0.786 (0.764–0.807)	0.914 (0.892–0.936)	0.871 (0.849–0.892)
Insomnia (*n* = 1999)	Sleep stage	0.953 (0.923–0.982)	0.989 (0.960–1.000)	0.964 (0.934–0.993)
	Arousal	0.805 (0.761–0.848)	0.907 (0.864–0.950)	0.892 (0.848–0.935)
Mild OSA (*n* = 4920)	Sleep stage	0.888 (0.872–0.903)	0.973 (0.957–0.989)	0.915 (0.899–0.930)
	Arousal	0.772 (0.744–0.799)	0.884 (0.856–0.911)	0.884 (0.856–0.911)
	Apnea	0.883 (0.855–0.910)	0.960 (0.932–0.987)	0.924 (0.896–0.951)
	Hypopnea	0.852 (0.824–0.879)	0.958 (0.930–0.985)	0.892 (0.864–0.919)
Moderate OSA (*n* = 4359)	Sleep stage	0.931 (0.913–0.948)	0.974 (0.956–0.991)	0.957 (0.939–0.974)
	Arousal	0.903 (0.875–0.930)	0.943 (0.915–0.970)	0.957 (0.927–0.986)
	Apnea	0.985 (0.955–1.000)	0.992 (0.962–1.000)	0.992 (0.962–1.000)
	Hypopnea	0.970 (0.942–0.997)	0.986 (0.958–1.000)	0.984 (0.956–1.000)
Severe OSA (*n* = 4443)	Sleep stage	0.787 (0.769–0.804)	0.955 (0.937–0.972)	0.833 (0.815–0.850)
	Arousal	0.735 (0.705–0.764)	0.853 (0.823–0.882)	0.881 (0.851–0.910)
	Apnea	0.912 (0.883–0.941)	0.962 (0.932–0.991)	0.948 (0.918–0.977)
	Hypopnea	0.833 (0.805–0.860)	0.942 (0.914–0.969)	0.880 (0.852–0.907)
Periodic leg movement disorder (*n* = 4848)	Sleep stage	0.927 (0.909–0.944)	0.988 (0.970–1.000)	0.939 (0.921–0.956)
	Arousal	0.830 (0.802–0.857)	0.935 (0.907–0.962)	0.893 (0.865–0.920)
	Periodic leg movement	0.789 (0.765–0.812)	0.897 (0.873–0.920)	0.909 (0.885–0.932)

OSA, obstructive sleep apnea.

## Data Availability

The datasets generated and/or analyzed during the current study can be accessed through the corresponding author upon reasonable request.

## References

[1] Kushida C.A., Littner M.R., Morgenthaler T., Alessi C.A., Bailey D., Coleman J., Friedman L., Hirshkowitz M., Kapen S., Kramer M. (2005). Practice parameters for the indications for polysomnography and related procedures: An update for 2005. Sleep.

[2] Stierer T., Punjabi N.M. (2005). Demographics and diagnosis of obstructive sleep apnea. Anesth. Clin. N. Am..

[3] Hornyak M., Feige B., Riemann D., Voderholzer U. (2006). Periodic leg movements in sleep and periodic limb movement disorder: Prevalence, clinical significance and treatment. Sleep Med. Rev..

[4] Jafari B., Mohsenin V. (2010). Polysomnography. Clin. Chest Med..

[5] Kakkar R.K., Hill G.K. (2007). Interpretation of the adult polysomnogram. Otolaryngol. Clin. N. Am..

[6] Hiensch R., Rapoport D.M. (2020). Sleep Studies Interpretation and Application. Otolaryngol. Clin. N. Am..

[7] Epstein L.J., Kristo D., Strollo P.J., Friedman N., Malhotra A., Patil S.P., Ramar K., Rogers R., Schwab R.J., Weaver E.M. (2009). Clinical guideline for the evaluation, management and long-term care of obstructive sleep apnea in adults. J. Clin. Sleep Med..

[8] Winkelman J.W., Berkowski J.A., DelRosso L.M., Koo B.B., Scharf M.T., Sharon D., Zak R.S., Kazmi U., Falck-Ytter Y., Shelgikar A.V. (2025). Treatment of restless legs syndrome and periodic limb movement disorder: An American Academy of Sleep Medicine clinical practice guideline. J. Clin. Sleep Med..

[9] Silber M.H., Ancoli-Israel S., Bonnet M.H., Chokroverty S., Grigg-Damberger M.M., Hirshkowitz M., Kapen S., Keenan S.A., Kryger M.H., Penzel T. (2007). The visual scoring of sleep in adults. J. Clin. Sleep Med..

[10] Malhotra R.K., Kirsch D.B., Kristo D.A., Olson E.J., Aurora R.N., Carden K.A., Chervin R.D., Martin J.L., Ramar K., Rosen C.L. (2018). Polysomnography for Obstructive Sleep Apnea Should Include Arousal-Based Scoring: An American Academy of Sleep Medicine Position Statement. J. Clin. Sleep Med..

[11] Troester M.M., Quan S.F., Berry R.B., Plante D.T., Abreu A.R., Alzoubaidi M., Bandyopadhyay A., DelRosso L., Ebben M., Kwon Y. (2023). The AASM Manual for the Scoring of Sleep and Associated Events: Rules, Terminology and Technical Specifications.

[12] Rosenberg R.S., Van Hout S. (2013). The American Academy of Sleep Medicine inter-scorer reliability program: Sleep stage scoring. J. Clin. Sleep Med..

[13] Rosenberg R.S., Van Hout S. (2014). The American Academy of Sleep Medicine Inter-scorer Reliability program: Respiratory events. J. Clin. Sleep Med..

[14] Deng S., Zhang X., Zhang Y., Gao H., Chang E.I., Fan Y., Xu Y. (2019). Interrater agreement between American and Chinese sleep centers according to the 2014 AASM standard. Sleep Breath.

[15] Magalang U.J., Arnardottir E.S., Chen N.H., Cistulli P.A., Gíslason T., Lim D., Penzel T., Schwab R., Tufik S., Pack A.I. (2016). Agreement in the Scoring of Respiratory Events Among International Sleep Centers for Home Sleep Testing. J. Clin. Sleep Med..

[16] Nikkonen S., Somaskandhan P., Korkalainen H., Kainulainen S., Terrill P.I., Gretarsdottir H., Sigurdardottir S., Olafsdottir K.A., Islind A.S., Óskarsdóttir M. (2024). Multicentre sleep-stage scoring agreement in the Sleep Revolution project. J. Sleep Res..

[17] Pitkänen H., Nikkonen S., Rissanen M., Islind A.S., Gretarsdottir H., Arnardottir E.S., Leppänen T., Korkalainen H. (2024). Multi-centre arousal scoring agreement in the Sleep Revolution. J. Sleep Res..

[18] Parrino L., Ferri R., Zucconi M., Fanfulla F. (2009). Commentary from the Italian Association of Sleep Medicine on the AASM manual for the scoring of sleep and associated events: For debate and discussion. Sleep Med..

[19] Reinke L., van der Heide E.M., Fonseca P., Absalom A.R., Tulleken J.E. (2025). Inter-rater disagreement in manual scoring of intensive care unit sleep data. BMC Res. Notes.

[20] Younes M., Raneri J., Hanly P. (2016). Staging Sleep in Polysomnograms: Analysis of Inter-Scorer Variability. J. Clin. Sleep Med..

[21] Lee Y.J., Lee J.Y., Cho J.H., Choi J.H. (2022). Interrater reliability of sleep stage scoring: A meta-analysis. J. Clin. Sleep Med..

[22] Altman D.G. (1990). Practical Statistics for Medical Research.

[23] Landis J.R., Koch G.G. (1977). The measurement of observer agreement for categorical data. Biometrics.

[24] Soh J.H., Kang Y.J., Yoon W.H., Park C.S., Shin H.W. (2024). Positional Obstructive Sleep Apnea and Periodic Limb Movements During Sleep: A Large Multicenter Study. Clin. Exp. Otorhinolaryngol..

